# Myocardium-Specific Deletion of Rac1 Causes Ventricular Noncompaction and Outflow Tract Defects

**DOI:** 10.3390/jcdd8030029

**Published:** 2021-03-15

**Authors:** Carmen Leung, Anish Engineer, Mella Y. Kim, Xiangru Lu, Qingping Feng

**Affiliations:** 1 Department of Physiology and Pharmacology, Schulich School of Medicine and Dentistry, Western University, London, ON N6A 5C1, Canada; carmen.leung73@gmail.com (C.L.); aengine@uwo.ca (A.E.); ykim655@uwo.ca (M.Y.K.); slu48@uwo.ca (X.L.); 2 Department of Medicine, Schulich School of Medicine and Dentistry, Western University, London, ON N6A 5C1, Canada

**Keywords:** *Rac1*, proliferation, cell polarity, congenital heart defects, compact myocardium, trabeculation

## Abstract

Background: Left ventricular noncompaction (LVNC) is a cardiomyopathy that can lead to arrhythmias, embolic events and heart failure. Despite our current knowledge of cardiac development, the mechanisms underlying noncompaction of the ventricular myocardium are still poorly understood. The small GTPase *Rac1* acts as a crucial regulator of numerous developmental events. The present study aimed to investigate the cardiomyocyte specific role of *Rac1* in embryonic heart development. Methods and Results: The *Nkx2.5-Cre* transgenic mice were crossed with *Rac1^f/f^* mice to generate mice with a cardiomyocyte specific deletion of *Rac1* (*Rac1^Nkx2.5^*) during heart development. Embryonic *Rac1^Nkx2.5^* hearts at E12.5–E18.5 were collected for histological analysis. Overall, *Rac1^Nkx2.5^* hearts displayed a bifid apex, along with hypertrabeculation and a thin compact myocardium. *Rac1^Nkx2.5^* hearts also exhibited ventricular septal defects (VSDs) and double outlet right ventricle (DORV) or overriding aorta. Cardiomyocytes had a rounded morphology and were highly disorganized, and the myocardial expression of Scrib, a planar cell polarity protein, was reduced in *Rac1^Nkx2.5^* hearts. In addition, cell proliferation rate was significantly decreased in the *Rac1^Nkx2.5^* ventricular myocardium at E9.5. Conclusions: *Rac1* deficiency in the myocardium impairs cardiomyocyte elongation and organization, and proliferative growth of the heart. A spectrum of CHDs arises in *Rac1^Nkx2.5^* hearts, implicating *Rac1* signaling in the ventricular myocardium as a crucial regulator of OFT alignment, along with compact myocardium growth and development.

## 1. Introduction

Congenital heart defects (CHDs) are the most common human birth defects, affecting up to 5% of live births [1,2]. The severity of these types of defects ranges from simple defects with no symptoms at birth, such as a small atrial septal defect (ASD), to complicated defects that are life-threatening and require intervention, such as Tetralogy of Fallot (TOF) [3,4]. One particular defect known as left ventricular noncompaction (LVNC) or “spongy myocardium” has no treatment at present, with the major therapy being heart failure and anticoagulation medications [5]. LVNC is classified as a rare genetic cardiomyopathy, occurring in 0.01% to 0.27% of the population, characterized by arrest of normal myocardium development, leading to a thin compact myocardial layer and an extensive non-compacted trabecular network [6,7]. Noncompaction of the ventricular myocardium can increase the risk of cardiac embolism, atrial fibrillation, ventricular arrhythmia and heart failure [5,7]. Although this defect occurs mostly in the LV, right ventricular (RV) noncompaction has also been reported in less than one-half of LVNC patients [8,9].

The ventricles grow and mature through a process of proliferation and differentiation to form trabeculation and compact myocardium [10]. In the mouse, trabecular formation begins at approximately E9.5 where cardiomyocytes grow to form protrusions of muscular ridges [11]. The trabeculae then undergo a process of remodeling/compaction where the bases of the trabeculation thicken and collapse into the myocardial wall. Proliferation of the compact myocardium is concomitant with remodeling. By E14.5, mature trabeculation is formed, along with a thick, compact myocardium [12,13]. Numerous signaling pathways have been implicated in development of the ventricular chambers including Notch, BMP, FGF, retinoic acid and planar cell polarity (PCP) signaling [10,14]. In addition, various mouse models of ventricular noncompaction defect have been generated but the ventricular defects between the mouse models are not consistent, reflecting the complex process of ventricular myocardium development. To date, the complete signaling mechanisms underlying ventricular myocardium development are still not completely understood [15,16].

The small GTPase, *Rac1*, acts as a crucial regulator of numerous developmental events including proliferation, cell cycle progression, cell survival, differentiation and regulation of cell shape, morphology and polarity [17]. Specifically, the importance of *Rac1* signaling in embryonic heart development has emerged in recent studies [18,19]. Our previous work has demonstrated a critical role of *Rac1* signaling in the anterior SHF and anterior-SHF derived structures including the RV, interventricular septum and the OFT. *Rac1* regulates cardiomyocyte polarization in the RV and formation of the cardiac apex [18]. In order to study the cardiomyocyte specific role of *Rac1* in heart development, the *Rac1* gene is deleted in the myocardium using the *Nkx2.5-Cre* transgenic mouse, which drives Cre recombinase activity in both the RV and LV [20]. We show that *Rac1* signaling is crucial for ventricular myocardium development and cardiomyocyte specific deficiency of *Rac1* leads to a spectrum of CHDs including a thin compact myocardium and hypertrabeculation, similar to clinical features of LVNC. *Rac1* deficient hearts also had a bifid cardiac apex and OFT alignment defects. Furthermore, we showed that a *Rac1* deficiency in the myocardium disrupts proliferation along with the organization and polarization of cardiomyocytes.

## 2. Methods

### 2.1. Mice

The *Rac1^f/f^* mouse line (Stock #5550) and *mT/mG* mouse line (Stock #7676) were purchased from Jackson Laboratory, Bar Harbor, ME, USA [21,22]. The *Nkx2.5-Cre* transgenic mouse was provided by Dr. Chi-Chung Hui at The Hospital for Sick Children, University of Toronto, originally generated by McFadden and colleagues [20]. The *mT/mG* mouse is a global double-fluorescent Cre reporter mouse. This reporter mouse expresses membrane-targeted Tomato (mT) before Cre-excision and membrane-targeted GFP (mG) after excision of mT [22]. A breeding program to generate *Nkx2.5-Cre;Rac1^f/f^* (*Rac1^Nkx2.5^*) and *Nkx2.5-Cre;mT/mG* mice was carried out and genotyping was performed as described previously [18]. Genotyping primer sequences are listed in Table 1. Mouse experiments and procedures were approved by the Animal Care Committee at Western University in accordance with the guidelines of the Canadian Council of Animal Care.

### 2.2. Histological Analysis

Embryonic samples (thoracic cavity) were fixed overnight in 4% paraformaldehyde at 4 °C, dehydrated and paraffin embedded. Samples were serially sectioned at 5 μm from the top of the aortic arch to the apex of the heart with a Leica RM2255 microtome. Sections were mounted onto positively charged albumin/glycerin coated microslides. Slides were stained with hematoxylin and eosin (H/E) for histological analysis and images were captured using a light microscope (Observer D1, Zeiss, Oberkochen, Germany).

### 2.3. Immunohistochemistry

Immunohistochemical staining was performed on paraffin heart sections. Antigen retrieval was carried out in sodium citrate buffer (pH 6.0) at 92 °C using a BP-111 laboratory microwave (Microwave Research & Applications, Carol Stream, IL, USA). Immunostaining was performed with primary antibodies for GFP (Abcam, Cambridge, MA, USA) and Scrib (Santa Cruz, Santa Cruz, CA, USA) followed by incubation with biotinylated secondary antibody and avidin and biotinylated HRP (Santa Cruz). 3-3′ diaminobenzidine tetrahydrochloride (DAB, Dallas, TX, USA) substrate solution was used to visualize the substrate and slides were counterstained with hematoxylin. Images were captured with Zeiss Observer D1 microscope using AxioVision Rel 4.7 software. For proliferation and apoptosis analysis, E9.5 heart samples were fixed in 4% paraformaldehyde for one hour, cryoprotected in 30% sucrose and embedded in FSC22 frozen section media (Leica, Wetzlar, Germany). Samples were sectioned in a sagittal orientation with a Leica cryostat at 10 μm thick onto glass slides. Slides were incubated with anti-phosphohistone-H3 (phospho S10) (Abcam), anti-cleaved caspase-3 (Cell Signaling) primary antibody, Alexa Fluor 647 wheat germ agglutinin (Invitrogen, Waltham, MA, USA), Alexa Fluor 488 phalloidin (Life Technologies, Carlsbad, CA, USA) and counterstained with Hoechst 33342 (Invitrogen). Confocal images were obtained at the Biotron Research Centre, Western University with a Zeiss LSM 510 Duo microscope. Cardiomyocyte cell size was assessed by measuring the cross-sectional diameter at the nuclear level in ~50 cardiomyocytes per section and 5 sections per heart in the LV free wall using AxioVision software (Zeiss, Oberkochen, Germany) [23].

### 2.4. Quantitative Real Time RT-PCR

Total RNA was isolated from E12.5 ventricular myocardium using the RNeasy Mini Kit (QIAGEN). Reverse transcription reaction was performed, as described previously [18]. Briefly, M-MLV Reverse Transcriptase (Invitrogen) and EvaGreen qPCR Mastermix (Abm, Vancouver, BC, Canada) were used for real time thermal cycling. 28S rRNA was used as an internal control. Samples were amplified for 35 cycles using the Eppendorf Mastercycler Realplex Real-Time PCR machine (Hamburg, Germany). Real time RT-PCR primer sequences are listed in Table 2. The mRNA level of *Rac1* in relation to 28S rRNA was determined using a comparative C_T_ method [24,25].

### 2.5. Western Blot Analysis

*Rac1* protein expression from E12.5 ventricular myocardium was measured by western blot analysis. Briefly, 25 μg of protein from isolated ventricular tissue was separated by 12% SDS-Page gel and transferred to nitrocellulose membranes. Blots were probed with antibodies against *Rac1* (1:500, Santa Cruz) and α-actinin (1:5000, Sigma, St. Louis, MO, USA). Blots were then washed and probed with horseradish peroxidase conjugated secondary antibodies (1:2500, Bio-Rad, Hercules, CA, USA) and detected using an ECL detection method. Densitometry was then performed to quantify the signal.

### 2.6. Statistical Analysis

Data are presented as means ± SEM. An unpaired Student’s *t* test was employed to determine statistical significance between the two groups. Differences were considered significant at *p* < 0.05.

## 3. Results

### 3.1. Generation of a Cardiomyocyte Specific Rac1 Knockout Mouse

**Figure 1:**
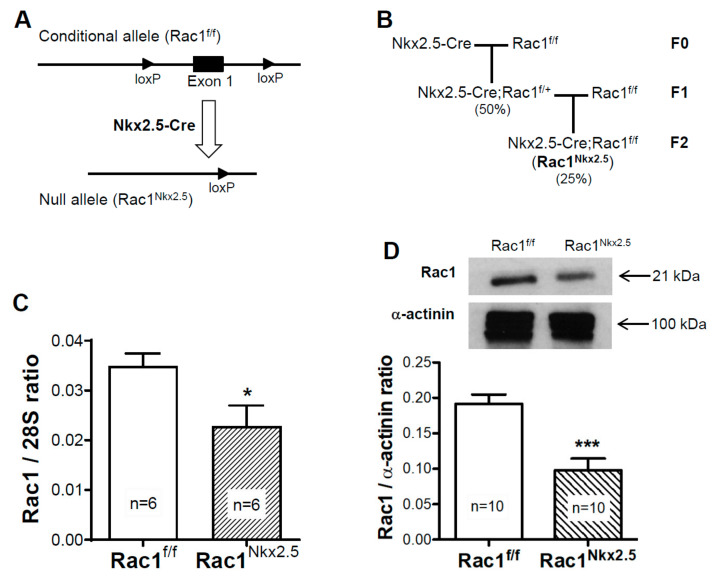
Generation of mouse line with a myocardium specific deletion of *Rac1* (*Rac1^Nkx2.5^*). A schema of the floxed *Rac1* allele and *Nkx2.5-Cre* mediated generation of the *Rac1* null allele (**A**). *Rac1^Nkx2.5^* mice were generated by crossing *Nkx2.5-Cre* transgenic mice with *Rac1^f/f^* mice for 2 generations with the expected genotype frequencies indicated in brackets (**B**). *Rac1* mRNA expression was significantly reduced in E12.5 *Rac1^Nkx2.5^* ventricular myocardium compared to *Rac1^f/f^* littermates (**C**). Western blot analysis of E12.5 ventricular myocardium showed a significant decrease in *Rac1* protein levels in *Rac1^Nkx2.5^* hearts compared to controls (**D**). * *p* < 0.05, *** *p* < 0.001 by unpaired Student’s *t*-test.

The Cre recombinase in the *Nkx2.5-Cre* mouse line is activated after E8.5 and initial specification of cardiac progenitors [20]. *Nkx2.5-Cre* transgenic mice and *Rac1^f/f^* mice were crossed to generate *Nkx2.5-Cre;Rac1^f/f^* (*Rac1^Nkx2.5^*) offspring (Figure 1A,B). To confirm a knockdown in *Rac1* mRNA expression, a real-time RT-PCR analysis was performed in RNAs isolated from E12.5 ventricles. The *Rac1* mRNA expression was significantly decreased by approximately 35% in *Rac1^Nkx2.5^* ventricular myocardium compared to littermate *Rac1^f/f^* controls (Figure 1C). *Rac1* protein levels in the ventricular myocardium were analyzed using western blotting. The ratio of *Rac1* to α-actinin protein levels was reduced by 49% in E12.5 *Rac1^Nkx2.5^* ventricular myocardium compared to littermate *Rac1^f/f^* controls (Figure 1D). These results confirm that *Nkx2.5-Cre*-mediated recombination sufficiently downregulates *Rac1* mRNA and protein expression in the ventricular myocardium of the developing heart.

### 3.2. Lineage Tracing of Nkx2.5-Cre Transgenic Mouse

**Figure 2:**
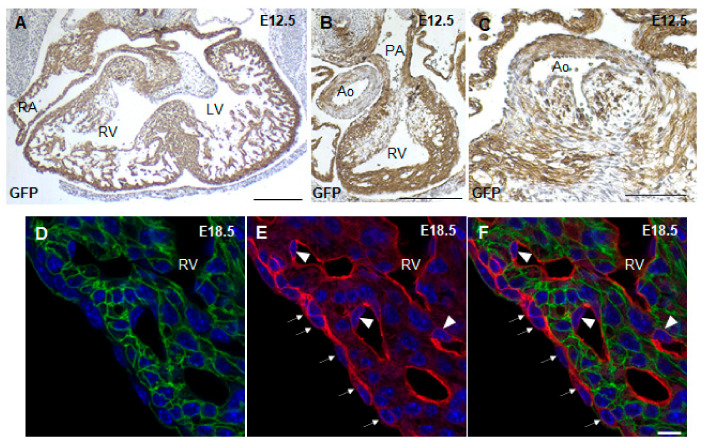
Lineage tracing with *Nkx2.5-Cre;mT/mG* mice. GFP immunostaining of E12.5 *Nkx2.5-Cre;mT/mG* paraffin heart sections showed Cre recombinase activity in the atrial and ventricular myocardium (**A**), OFT (**B**) and aortic valve leaflets (**C**). Fluorescence imaging of cryosections of E18.5 *Nkx2.5-Cre;mT/mG* hearts showed that the epicardium (arrows) and endothelial cells (arrowhead) remain RFP^+^ in *Nkx2.5-Cre;mT/mG* hearts (**D**–**F**). RV, right ventricle; LV, left ventricle; PA, pulmonary artery; Ao, aorta; epi, epicardium. Scale bars: 250 µm (**A**–**C**), 10 µm (**D**–**F**).

To trace where the Cre recombinase is active in *Nkx2.5-Cre* transgenic hearts, *Nkx2.5-Cre* mice were crossed to *mT/mG* reporter mice, which marks all tissues possessing Cre recombinase activity with GFP. McFadden et al., the group who first created the *Nkx2.5-Cre* transgenic mouse, used a lacZ reporter to show that the Cre recombinase is active throughout the ventricular myocardium with minimal recombination in the OFT and atria [20]. Using the *Nkx2.5-Cre;mT/mG* mouse, we showed that the Cre recombinase was active throughout the ventricular myocardium and a large portion of the atria at E12.5 (Figure 2A). In addition, a majority of the pulmonary artery myocardium and part of the aorta were GFP^+^ in *Nkx2.5-Cre;mT/mG* hearts (Figure 2B). Closer analysis of the aortic valves showed that some of the cells in the early aortic valves were also GFP^+^ in *Nkx2.5-Cre;mT/mG* hearts (Figure 2C), indicating the contribution of *Nkx2.5* expressing cells to the development of the OFT and atria. Furthermore, closer examination of the epicardium and endocardial cells at E18.5 showed that these cells remained RFP^+^, indicating no Cre recombinase activity in these cell types (Figure 2D–F). Thus, the *Nkx2.5-Cre* mouse drives recombination in the ventricular myocardium, atria and part of the OFT.

### 3.3. Congenital Heart Defects in Rac1^Nkx2.5^ Mice

**Figure 3:**
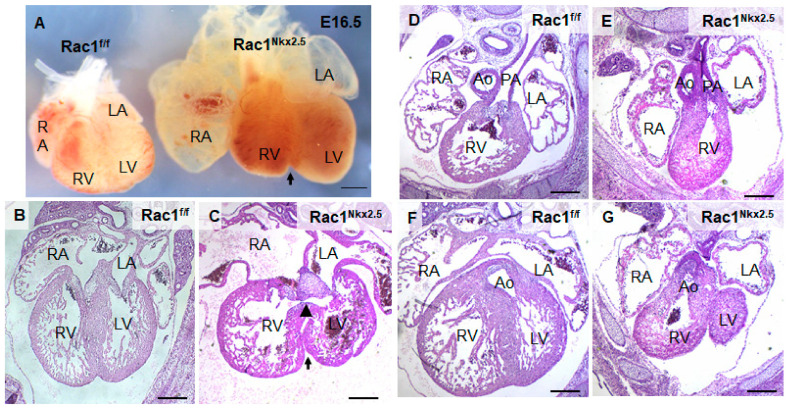
Congenital heart defects in *Rac1^Nkx2.5^*. (**A**) Bifid cardiac apex in an E16.5 *Rac1^Nkx2.5^* heart. Arrow indicates bifurcation between the RV and LV. (**B**,**C**) Ventricular septal defect (arrowhead) was found in E15.5 *Rac1^Nkx2.5^* hearts with an arrow indicating the bifid cardiac apex. (**D**–**G**) Double outlet right ventricle (DORV) was found in E15.5 *Rac1^Nkx2.5^* hearts. The pulmonary artery and aorta were connected to the RV and LV, respectively in *Rac1^f/f^* control hearts (**D**,**F**). However, both pulmonary artery and aorta were connected to the RV in *Rac1^Nkx2.5^* hearts (**E**,**G**). RA, right atrium; LA, left atrium. Scale bars: 500 µm.

**Figure 4:**
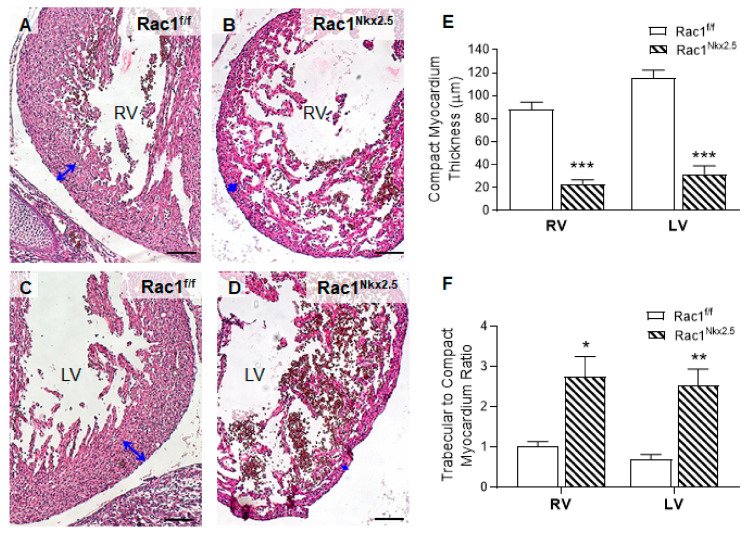
Ventricular myocardium abnormalities in *Rac1^Nkx2.5^* hearts at E15.5. In comparison to *Rac1^f/f^* controls (**A**,**C**), the *Rac1^Nkx2.5^* mice (**B**,**D**) show thin compact myocardium and hypertrabeculation in the RV and LV wall. The thickness of the compact myocardium in *Rac1^Nkx2.5^* hearts (*n* = 5) was decreased while trabecular to compact myocardium ratio was increased as compared to littermate controls (*n* = 6) (**E**,**F**). Double-headed arrows in (**A**–**D**) indicate measurements of compact myocardium thickness. * *p* < 0.05, ** *p* < 0.01, *** p< 0.001 vs. *Rac1^f/f^* by Student’s *t*-test. Scale bars: 100 µm.

*Rac1^Nkx2.5^* embryos were alive from E11.5–18.5, but all neonates were found dead at P0 (*n* = 5). Gross morphological analysis of these P0 *Rac1^Nkx2.5^* hearts revealed a bifid cardiac apex, similar to what was observed and reported in our previous study with a *Mef2c-Cre* anterior second heart field-specific deletion of *Rac1* [18]. Examination of all *Rac1^Nkx2.5^* hearts at earlier embryonic time points showed evidence of a bifid cardiac apex as well (Figure 3A). In addition, *Rac1^Nkx2.5^* hearts had incomplete development of the interventricular septum, resulting in a ventricular septal defect (VSD) (Figure 3B,C, Table 3). Alignment of the outflow tract (OFT) to the ventricles was also defective in *Rac1^Nkx2.5^* hearts compared to littermate *Rac1^f/f^* controls. A double outlet right ventricle (DORV) was observed in 11 of the 17 *Rac1^Nkx2.5^* hearts (Figure 3D–G, Table 3) and 6 of the 17 *Rac1^Nkx2.5^* hearts exhibited an overriding aorta (Table 3). In addition, both the left ventricle (LV) and right ventricle (RV) of *Rac1^Nkx2.5^* hearts show a thin compact myocardium and hypertrabeculation (Figure 4A–D). The compact myocardium of both *Rac1^Nkx2.5^* ventricles at E15.5 was poorly formed and significantly thinner while the trabecular to compact myocardium ratio increased by more than 2.5-fold compared to littermate *Rac1^f/f^* controls (Figure 4E,F). These findings suggest a critical role for *Rac1* in interventricular septum formation, OFT alignment and development of the trabecular and compact ventricular myocardium.

### 3.4. Loss of F-Actin Filament Organization and Cardiomyocyte Polarity in Rac1^Nkx2.5^ Hearts

**Figure 5:**
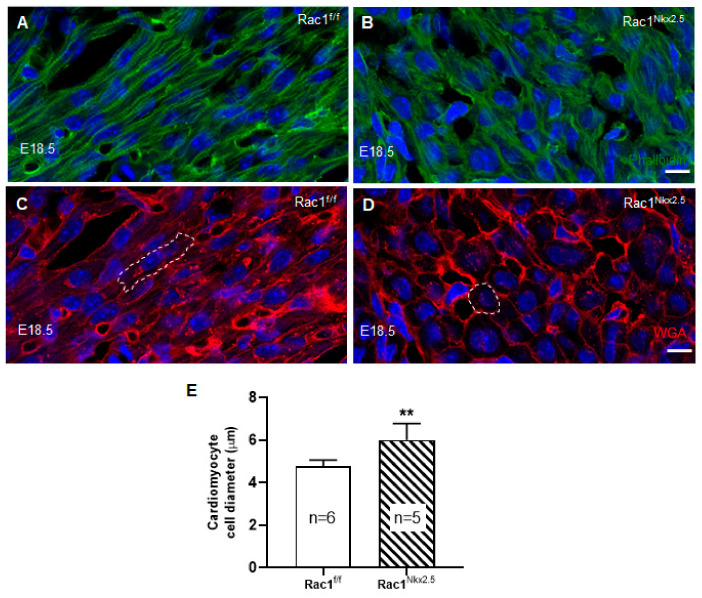
Disruption of F-actin organization and cardiomyocyte polarity in *Rac1^Nkx2.5^* hearts. E18.5 cryosections of the heart were double stained for phalloidin and wheat germ agglutinin (WGA) to assess F-actin organization and cardiomyocyte cell polarity, respectively. The images were taken from the corresponding areas of LV myocardium free wall in *Rac1^f/f^* and *Rac1^Nkx2.5^* mice. F-actin filament organization was disrupted in *Rac1^Nkx2.5^* myocardium (**B**) compared to littermate controls (**A**). WGA staining shows rounded cardiomyocytes in *Rac1^Nkx2.5^* ventricular myocardium (**D**) compared to the elongated cardiomyocytes in littermate controls (**C**). The cell borders of a cardiomyocyte in (**C**,**D**) are outlined. The short axis of cardiomyocyte diameter was significantly larger in *Rac1^Nkx2.5^* compared to littermates (**E**). ** *p* < 0.01 by unpaired Student’s *t*-test. Scale bars: 10 µm.

To analyze F-actin filament organization and cardiomyocyte polarity in the ventricular myocardium, E18.5 *Rac1^Nkx2.5^* heart sections were double stained with phalloidin and wheat germ agglutinin (WGA) to mark F-actin filaments and cell borders, respectively. Our data show that *Rac1^Nkx2.5^* hearts had severely disrupted F-actin filament organization compared to controls, which had long, parallel running F-actin filaments throughout the myocardium (Figure 5A,B). Additionally, WGA staining revealed rounded, spherically shaped cardiomyocytes in *Rac1^Nkx2.5^* hearts, in both the RV and LV (Figure 5D). In comparison, littermate ventricular myocardium had cardiomyocytes that underwent polarization with an elongated shape and were well organized/aligned in both the RV and LV (Figure 5C). Furthermore, quantitative analysis revealed that the cardiomyocyte cell size was significantly larger in *Rac1^Nkx2.5^* compared to the littermate controls (Figure 5E). This data suggests a crucial role for *Rac1* in F-actin filament organization, polarization and elongation of cardiomyocytes during embryonic heart development.

### 3.5. Decreased Scrib Protein Expression in Rac1^Nkx2.5^ Hearts

**Figure 6:**
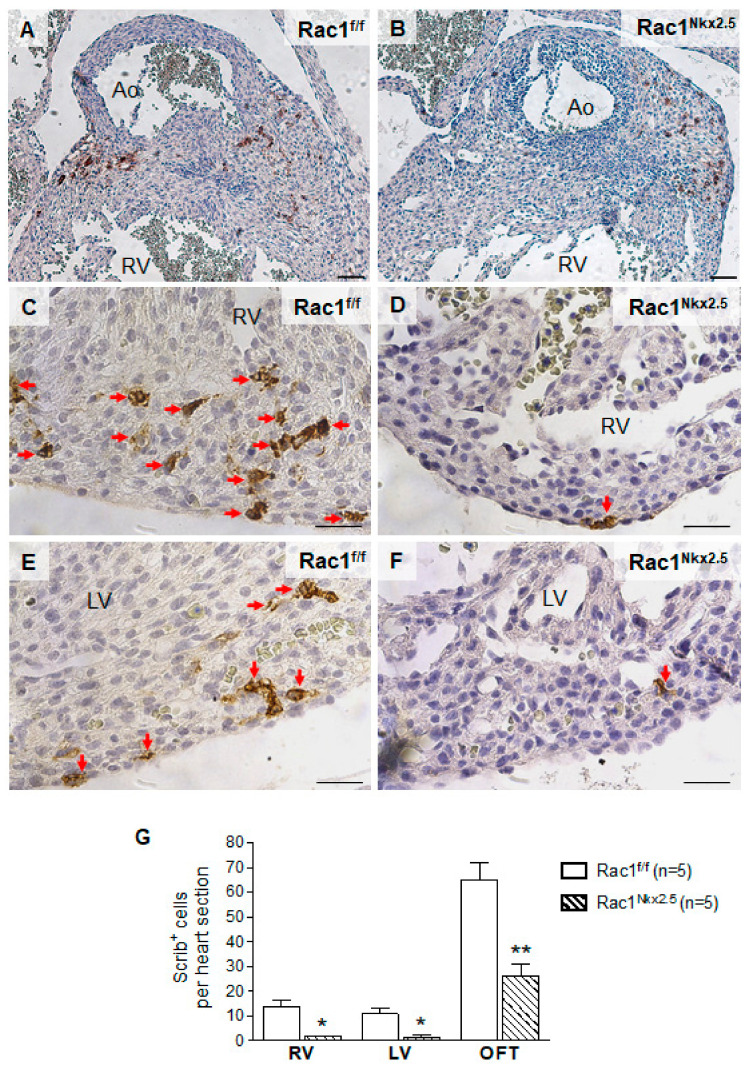
Loss of Scrib expression in *Rac1^Nkx2.5^* hearts. Scrib immunostaining was performed on E15.5 *Rac1^Nkx2.5^* and *Rac1^f/f^* heart sections. The areas analyzed included the myocardium surrounding the aorta (**A**,**B**), RV (**C**,**D**) and the LV (**E**,**F**). Five sections per heart were used. The number of Scrib-expressing positive cells was significantly decreased in the myocardium surrounding the aorta (OFT), RV and LV of *Rac1^Nkx2.5^* hearts (**G**). * *p* < 0.05, ** *p* < 0.01 by Student’s *t*-test. Scale bars: 50 µm.

Scrib plays an important role in cell polarity through interacting with *Rac1* in the developing myocardium. Loss of either *Scrib* or *Rac1* leads to a reduction in membrane association of the other [19]. To analyze the expression of Scrib, immunostaining was performed on E15.5 *Rac1^Nkx2.5^* hearts. Scrib was highly expressed in the myocardium surrounding the opening of the aorta in control E15.5 hearts (Figure 6A). In comparison, the expression of Scrib in this area was reduced in E15.5 *Rac1^Nkx2.5^* heart sections (Figure 6B). Similar to what was described in our previous study [18], Scrib protein expression was abundant in the interventricular junction in control hearts at E15.5. However, expression of Scrib was reduced in E15.5 *Rac1^Nkx2.5^* the myocardium of interventricular junction compared to littermate controls (Figure 6C–F). Overall, Scrib protein expression was significantly decreased in E15.5 *Rac1^Nkx2.5^* hearts compared to littermate controls (Figure 6G). The loss of Scrib expression in *Rac1^Nkx2.5^* hearts suggests a disruption in cell polarity and the PCP pathway, further supporting a failure of cardiomyocytes to undergo polarization.

### 3.6. Decreased Cell Proliferation in Rac1^Nkx2.5^ Hearts

**Figure 7:**
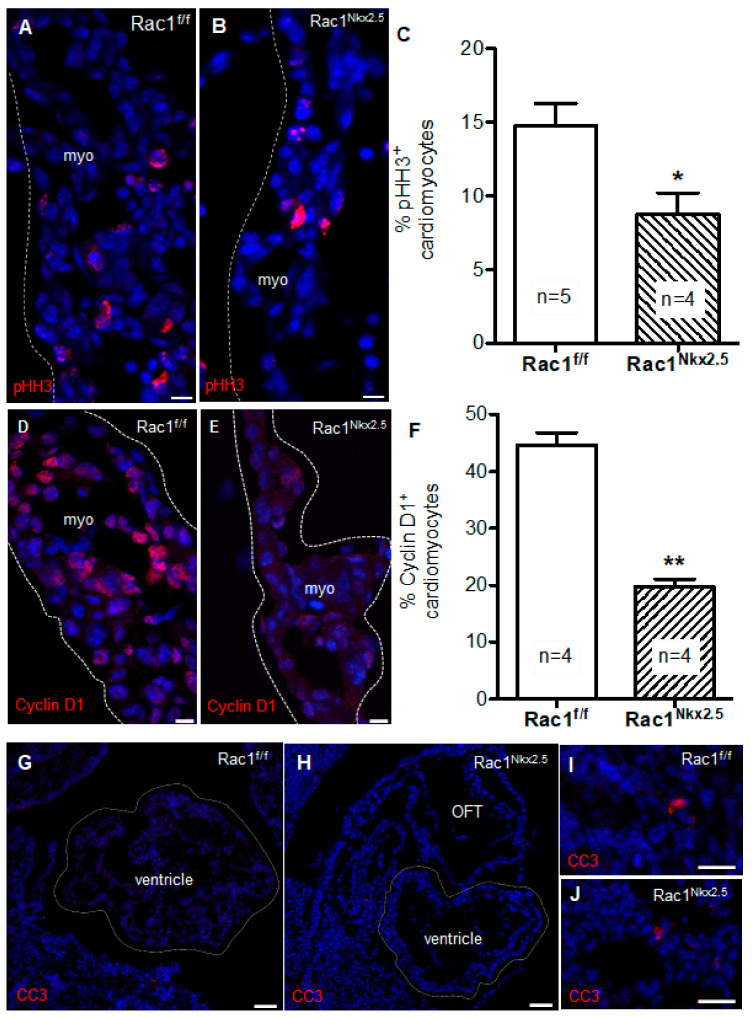
Decreased proliferation rate in *Rac1^Nkx2.5^* hearts. Phospho-histone H3 (pHH3) immunostaining to mark proliferating cells undergoing mitosis in ventricular myocardium (myo) of E9.5 *Rac1^Nkx2.5^* and *Rac1^f/f^* hearts (**A**,**B**). Proliferation rate was significantly decreased in E9.5 *Rac1^Nkx2.5^* ventricular myocardium compared to littermate controls (**C**). Cyclin D1 immunostaining in E9.5 *Rac1^f/f^* and *Rac1^Nkx2.5^* ventricular myocardium marked cell progression through G1 (**D**,**E**). Cyclin D1 expression was significantly decreased in E9.5 *Rac1^Nkx2.5^* ventricular myocardium compared to littermate controls (**F**). Cleaved caspase-3 (CC3) immunostaining to mark apoptotic cells in ventricular myocardium of E9.5 *Rac1^Nkx2.5^* and *Rac1^f/f^* hearts. No apoptosis was detected in E9.5 *Rac1^Nkx2.5^* and *Rac1^f/f^* ventricular myocardium (**G**,**H**). Apoptotic cells were detected in tissues outside of the heart in *Rac1^f/f^* and *Rac1^Nkx2.5^* embryos (**I**,**J**). * *p* < 0.05, ** *p* < 0.01 by unpaired Student’s *t*-test. Scale bars: 10 µm (**A**,**B**), 50 µm (**D**–**G**).

The observed defects in *Rac1^Nkx2.5^* ventricular myocardium development could also be attributed to a decrease in cell proliferation and/or aberrant apoptosis. Cell proliferation has been shown to be highest at E9.5 in the developing mouse heart [26]. Thus, proliferation of E9.5 *Rac1^Nkx2.5^* hearts was analyzed by immunostaining for phospho-histone H3 (pHH3) and cyclin D1, which are a marker of the mitotic phase of cell division and a cell cycle regulator, respectively. The cell proliferation rate assessed by pHH3^+^ and cyclin D1^+^ cells in the ventricular myocardium was significantly reduced in E9.5 *Rac1^Nkx2.5^* hearts compared to littermate *Rac1^f/f^* controls (Figure 7A–F). Immunostaining for cleaved caspase-3 (CC3), a marker of activated apoptosis, showed little to no apoptosis in both control E9.5 and *Rac1^Nkx2.5^* ventricular myocardium (Figure 7G,H). However, apoptosis was detected in tissues outside the heart (Figure 7I,J). The decreased proliferation rate in *Rac1^Nkx2.5^* hearts suggests a critical role for *Rac1* in regulating cardiomyocyte proliferation in the ventricular myocardium, after initial specification of cardiac progenitors.

### 3.7. Decreased Scrib and Cardiac Transcription Factor Expression in Rac1^Nkx2.5^ Hearts

**Figure 8:**
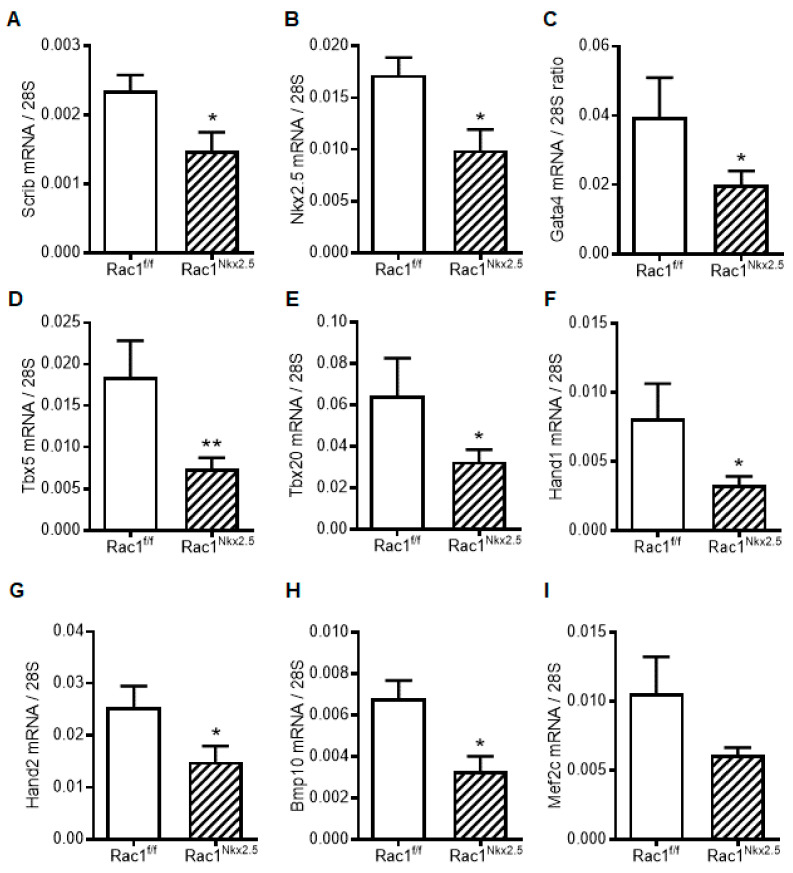
Myocardial mRNA expression of transcription and growth factors in E12.5 *Rac1^f/f^* and *Rac1^Nkx2.5^* hearts. (**A**) *Scrib*. (**B**) *Nkx2.5*. (**C**) *Gata4*. (**D**) *Tbx5*. (**E**) *Tbx20*. (**F**) *Hand1*. (**G**) *Hand2*. (**H**) *Bmp10*. (**I**) *Mef2c*. Data are expressed as mRNA to 28S ratios. *n* = 5–6 per group. * *p* < 0.05, ** *p* < 0.01 by unpaired Student’s *t* test.

To assess the genetic pathways regulated by *Rac1* signaling, we analyzed the mRNA expression of factors critical to embryonic heart development in E12.5 hearts. Since Scrib-*Rac1* interaction is crucial for normal heart development, *Scrib* mRNA levels were assessed. Consistent with its protein expression (Figure 6), *Scrib* mRNA levels were significantly reduced in E12.5 *Rac1^Nkx2.5^* compared to *Rac1^f/f^* hearts (Figure 8A). Additionally, cardiac transcription and growth factors including *Nkx2.5*, *Gata4*, *Tbx5*, *Tbx20*, *Hand1*, *Hand2* and *Bmp10*, except *Mef2c,* were all significantly decreased in E12.5 *Rac1^Nkx2.5^* compared to *Rac1^f/f^* hearts (Figure 8B–I). These results indicate that transcriptional regulation of heart development was severely disrupted in the *Rac1^Nkx2.5^* hearts.

## 4. Discussion

**Figure 9:**
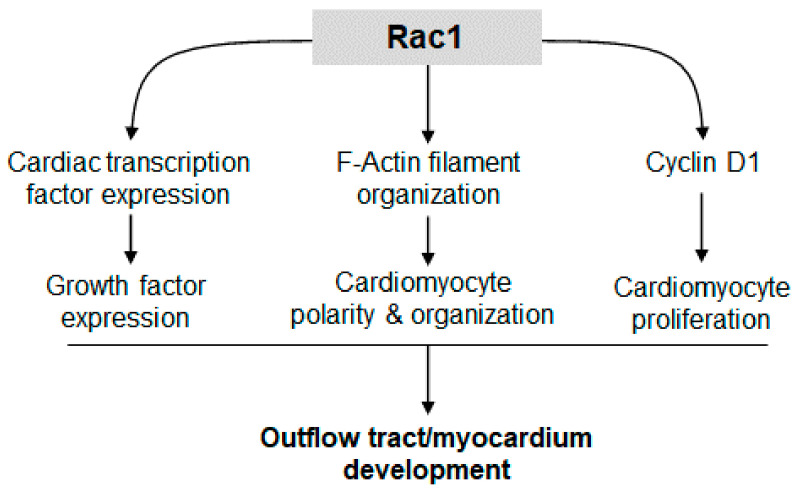
*Rac1* signaling promotes cardiac transcription/growth factor expression, cardiomyocyte polarity and proliferation, leading to normal development of the outflow tract and compact myocardium.

Recent studies have implicated PCP signaling and cell polarity as critical regulators of compact myocardium development. *Vangl2, Scrib* and *Dishevelled* mouse mutants have cardiomyocytes that are not polarized and the ventricular myocardium is thinned, resembling LVNC [27,28,29]. *Rac1* is a known downstream effector of PCP signaling; however, the cardiomyocyte specific role of *Rac1* in heart development is unclear [30]. In the present study, we demonstrated that downregulation of *Rac1* signaling in the ventricular myocardium disrupted formation of a trabecular network and development of the compact myocardium. In addition, *Rac1^Nkx2.5^* mice had a bifid cardiac apex, defects in ventricular septum formation and OFT alignment. The F-actin filament organization and polarization of cardiomyocytes in the *Rac1* deficient ventricular myocardium was also abnormal. Overall, cell proliferation was decreased in *Rac1^Nkx2.5^* hearts, along with expression of the PCP protein, Scrib. Our study demonstrates a critical role for *Rac1* signaling in outflow tract and compact myocardium development (Figure 9).

Cell proliferation is a regulated spatially and temporally during heart development. The rate of cell proliferation in the ventricular myocardium peaks at E9.5 and gradually decreases during development [26]. Studies have shown that cardiomyocyte proliferation is the major determinant of overall cardiac size during heart development. The mass of the heart must increase to match the increasing circulatory demands of the growing embryo [31]. *Rac1* has been shown to regulate cell proliferation through various pathways. Our data showed that a *Rac1* deficiency in the ventricular myocardium decreased the expression of factors critical to cardiogenesis, cardiomyocyte differentiation and proliferation including *Nkx2.5*, *Gata4*, *Tbx5*, *Tbx20*, *Hand 1*, *Hand2* and *Bmp10*. Additionally, Cyclin D1 protein levels and cell proliferation were reduced in E9.5 *Rac1^Nkx2.5^* hearts. These results suggest that *Rac1* promotes cardiac transcription and growth factor expression, leading to cell cycle progression and cardiomyocyte proliferation in the developing heart (Figure 9).

An earlier report by Boczonadi et al. also used a *Nkx2.5-Cre;Rac1^f/f^* mouse line in their studies [19]. However, the *Nkx2.5-Cre* mouse used by Boczonadi et al. was an *Nkx2.5* heterozygous mouse since the Cre recombinase gene was knocked into the *Nkx2.5* genetic locus [32]. In contrast, the *Nkx2.5-Cre* mouse used in the present study is a transgenic mouse and retains two intact alleles of *Nkx2.5* [20]. The *Nkx2.5-Cre;Rac1^f/f^* mice used in Boczonadi et al. were embryonically lethal due to their interaction with E13.5, which precluded analysis of ventricular septation and OFT alignment with the developing ventricles [19]. However, at E12.5 these mice did show underdeveloped ventricles with a thin ventricular wall. The earlier lethality of the *Nkx2.5-Cre;Rac1^f/f^* mouse line compared to the *Rac1^Nkx2.5^* used in the current study is likely due to the heterozygous expression of *Nkx2.5* compounded with decreased *Rac1* signaling in the ventricular myocardium. Furthermore, whether the underdeveloped heart reported in the study by Boczonadi et al. [19] is exclusively due to deficient *Rac1* signaling or also due to downregulation of *Nkx2.5* is unclear. Our data implicates *Rac1* signaling specifically and we were able to demonstrate that *Rac1^Nkx2.5^* hearts exhibit VSDs and OFT alignment defects.

Molecular players in the planar cell polarity (PCP) pathway are not only critical for normal myocardial development but also govern OFT development. For example, mutation in *Vangl2*, a member of the highly conserved non-canonical Wnt signaling cascade, results in cardiac malalignment and DORV [28]. In addition, *Dishevelled 2* knockout mice show transposition of the great arteries, persistent truncus arteriosus and DORV, with the latter being the most common cardiac defect [33]. Notably, anterior SHF specific deletion of *Rac1* results in a spectrum of OFT alignment defects including DORV via a disruption of migration of neural crest cells and cardiomyocytes into the OFT [34]. In the present study, *Nkx2.5-Cre-driven* GFP expression is seen in pulmonary and aortic walls, and in aortic valve leaflets, suggesting cardiomyocyte migration to the OFT. The OFT myocardium produces axon guidance molecules such as Sema3c, a chemoattractant that navigates neural crest cells to the OFT [34]. Reductions in cardiomyocyte proliferation and migration to the OFT may diminish cardiomyocyte contribution to the OFT and impede neural crest cell migration, leading to OFT alignment defects.

Our previous work reported a bifid cardiac apex when *Rac1* was specifically deleted in the anterior SHF and all anterior SHF-derived cells [18]. We had postulated that loss of *Rac1* signaling in the RV led to an inability of the nonpolarized and disorganized cardiomyocytes to bridge the interventricular junction to unify the two developing ventricles and form a distinct cardiac apex. However, in the present study using the *Rac1^Nkx2.5^* hearts, we show that development of normal cardiac apex is also dependent on polarity and organization of the cardiomyocytes in the LV myocardium. Interestingly, other reports where PCP signaling was disrupted did not report observations of a bifid cardiac apex, despite similar reports of cardiomyocyte disorganization [27,28]. *Rac1* is a known downstream effector of PCP signaling, regulating actin cytoskeleton dynamics and cell polarity [35,36]. Our findings suggest that development of a bifid cardiac apex involves a pathway specific to a disruption of *Rac1* signaling. In addition, since *Rac1* is a pleiotropic effector of numerous cellular events, the concomitant disruption of several cellular mechanisms is likely responsible for bifid cardiac apex, along with the other observed CHDs in *Rac1^Nkx2.5^* hearts.

Our lineage tracing analysis with the *mT/mG* global double florescence mouse showed similar GFP^+^ expression in domains of the heart that were reported by McFadden et al, who used a lacZ reporter. However, we also showed Cre recombinase activity in the OFT and atria, which were reported to be minimal by McFadden et al [20]. This discrepancy in Cre recombinase activity may be due to the additional β-galactosidase enzymatic reaction step that is required to visualize the blue color of lacZ staining, making it a less efficient reporter compared to a GFP reporter. These results suggest that future lineage tracing analysis should use a florescence reporter as a more reliable readout of expression compared to a lacZ reporter.

*Rac1* is involved in reactive oxygen species (ROS) generation through activation of NADPH oxidase [37]. ROS mediates numerous cellular functions including proliferation, cell survival, differentiation and migration [37,38]. The levels of ROS are finely tuned in a cell to regulate these diverse functions. For example, excess ROS induces apoptosis while basal levels of ROS regulate gene expression and proliferation [37,39]. Work in our lab has shown that ROS levels must be tightly regulated to facilitate normal cardiac development. Excess ROS induced by pregestational maternal diabetes and decreased levels of ROS in the NADPH oxidase *Nox2* knockout mouse both have a detrimental effect on heart development, leading to a spectrum of CHDs [40,41,42]. Whether the CHDs observed in the *Rac1^Nkx2.5^* hearts can also be attributed to decreased ROS levels should be determined in future studies.

## 5. Conclusions

A *Rac1* deficiency in the myocardium disrupts cardiomyocyte organization and proliferation, leading to bifid cardiac apex, VSDs and OFT alignment and ventricular myocardial compaction defects. Our study suggests a critical role for *Rac1* regulation of cardiomyocyte proliferation, organization and polarization in development of the outflow tract and ventricular myocardium. Whether perturbed *Rac1* signaling in the ventricular myocardium underlies human cases of LVNC warrants further investigation.

## Figures and Tables

**Table 1:** PCR primer sequences for genotyping genetically modified mice.

Mice	Forward (5′-3′)	Reverse (5′-3′)
*Nkx2.5-Cre*	TGCCACGACCAAGTGACAGC	CCAGGTTACGGATATAGTTCATG
*Rac1^f/f^*	TCCAATCTGTGCTGCCCATC	GATGCTTCTAGGGGTGAGCC
*mTmG*	CTCTGCTGCCTCCTGGCTTCT	CGAGGCGGATCACAAGCAATA Mutant reverse: TCAATGGGCGGGGGTCGTT

**Table 2:** Primer sequences for real time RT-PCR analysis.

Gene	Forward (5′-3′)	Primer Sequence (5′-3′)
*Rac1*	TTGTCCAGCTGTGTCCCATA	AACCTGCCTGCTCATCAGTT
*Gata4*	GCCTGCGATGTCTGAGTGAC	CACTATGGGCACAGCAGCTC
*Nkx2.5*	GACAGCGGCAGGACCAGACT	CGTTGTAGCCATAGGCATTG
*Tbx5*	AGGAGCACAGTGAGGCACAA	GGGCCAGAGACACCATTCTC
*Tbx20*	CACCTATGGGGAAGAGGATGTTC	GTCGCTATGGATGCTGTACTGGT
*Mef2c*	TACCCCGGTGGTTTCCGTAG	CCCAACTGACTGAGGGCAGA
*Scrib*	AGGAGGAGAACAGGGATGAGGAG	CCTTTGTAGGGGGTAGAGCCTTT
*Bmp10*	CCACTCGGATCAGGAGGAAC	CACACAGCAGGCTTTGGAAG
*Hand1*	TGGCTACCAGTTACATCGCCTAC	GTGCGCCCTTTAATCCTCTTCT
*Hand2*	GCTACATCGCCTACCTCATGGAT	TCTTGTCGTTGCTGCTCACTGT
*28S*	ACATTGTTCCAACATGCCAG	TTGAAAATCCGGGGGAGAG

**Table 3:** Congenital heart defects in *Rac1^Nkx2.5^* mice (E14.5—P0).

	Bifid Apex	VSD	DORV	Overriding Aorta	Thin Compact Myocardium
N = 17	17	17	11	6	17
%	100	100	64.7	35.3	100

VSD, ventricular septal defect; DORV, double outlet right ventricle. All 17 *Rac1^SHF^* hearts had more than one type of CHD. No CHDs defects were found in littermate E14.5-P0 *Rac1^f/f^* hearts (*n* = 11).

## Data Availability

The data that support the findings of this study are available from the corresponding author upon reasonable request.
